# Effects of 7-day polyphenol powder supplementation on cycling performance and lung function in an ozone-polluted environment

**DOI:** 10.1007/s00421-023-05287-0

**Published:** 2023-07-30

**Authors:** Lillian C. Morton, Carl D. Paton, Troy Merry, Andrea J. Braakhuis

**Affiliations:** 1 Department of Nutrition, Faculty of Medical and Health Science, The University of Auckland, Auckland, New Zealand; 2 School of Health and Sport Science, The Eastern Institute of Technology, Napier, New Zealand

**Keywords:** Ozone, Cycling performance, Polyphenols, Respiratory

## Abstract

**Purpose:**

Polluted environments can adversely affect lung function and exercise performance. Evidence suggests that some nutrient supplements may offset pollution’s detrimental effects. This study examined the effect of polyphenol supplementation on lung function and exercise performance in an ozone-polluted environment.

**Methods:**

Ten male cyclists (mean ± SD: age, 43.8 ± 12.4 years; height, 177.8 ± 7.1 cm; weight, 76.03 ± 7.88 kg; VO_2max_ 4.12 ± 0.72 L min^−1^) initially completed a baseline maximal incremental test and maximal effort 4 km time trial in ambient air. Thereafter cyclists completed two trials in an ozone-polluted environment (0.25 ppm) following seven days of supplementation with either polyphenol (PB) or placebo (PL). Experimental trials consisted of a three-stage submaximal test (50%, 60% and 70% incremental peak power) followed by a 4 km time trial. Lung function was measured pre- and post-exercise via spirometry and adverse respiratory symptoms with a Likert scale.

**Results:**

Ozone exposure significantly reduced (*p* < 0.05) lung function relative to ambient air. There were no significant differences (*p* > 0.05) in measured variables across the three submaximal intensities. There was a small (*d* = 0.31) non-significant difference (*p* = 0.09) in 4 km performance in PB (406.43 ± 50.29 s) vs. PL (426.20 ± 75.06 s). Oxygen consumption during the time trial was greater in PB (3.49 ± 0.71 L min^−1^) vs PL (3.32 ± 0.71 L min^−1^, *p* = 0.01, *d* = 0.24). Cough severity (SOC) was lower (*p* = 0.03) with PB relative to PL.

**Conclusion:**

PB supplementation may provide small benefits to performance and reduce cough symptoms during high-intensity exercise in ozone-polluted environments.

## Introduction

Ozone is a principal component of photochemical air pollution endogenous to numerous metropolitan areas and is a potent oxidant gas. Ozone exposure is known to induce irritant effects on the respiratory tract impairing lung function, resulting in subjective symptoms of respiratory discomfort and detriments in exercise capacity and performance in susceptible and healthy individuals (Adams 1987; Bosson et al. 2008).

Athletes often have greater exposure to air pollutants when training outdoors and may be more susceptible to its negative effects (Carlisle and Sharp 2001). During exercise there is an increase in minute ventilation, and at higher-exercise intensities, a larger fraction of air is inhaled which will proportionately increase the quantity of pollutants. The air flow velocity of this higher respiration also carries the pollutants deeper into the respiratory tract (Carlisle and Sharp 2001; Kubesch et al. 2015). Research examining the relationship between ozone exposure, athletic performance and lung function reports that both aerobic capacity and lung function are negatively affected by poor air quality and ozone exposure (Foxcroft and Adams 1986; Reche et al. 2020), and that continuous exercise produces a greater effect (Adams 1987).

Acute airway epithelial inflammatory response to ozone results in bronchial airway narrowing, reduced pulmonary gas diffusion and arterial oxygenation, impaired vasoregulation, and increased blood viscosity (Cakmak et al. 2011). The pathways underlying these effects are complex and poorly understood; however, oxidative stress repeatedly emerges as a potential mechanism in all these detrimental cardiovascular and respiratory actions. Ozone inhalation induces the production of reactive oxygen species (ROS) within the airways by interacting with the epithelial lining fluid and unsaturated compounds present in the respiratory tract (Gomes et al. 2011, Cho et al. 2006, Mudway 1999), activating ROS-signalling pathways and initiating inflammatory responses (Cho and Kleeberger 2010; Holz et al. 1999; Mumby et al. 2019).

Large-scale epidemiological studies have found a negative correlation between polyphenol consumption and respiratory disease, respiratory decline and prevalence of respiratory symptoms (Nyanhanda et al. 2014; Garcia-Larsen et al. 2015, Garcia-Larsen et al. 2018, Morton and Braakhuis 2021). A sub-class of polyphenols, anthocyanins and proanthocyanins, have been found to attenuate lung inflammation (Nyanhanda et al. 2014, Shaw et al. 2020). Blackcurrants (*Ribes nigrum L*.) are very high in antioxidant activity and are amongst the top dietary sources of anthocyanins. Blackcurrant (BC) supplementation has been shown to improve cycling performance under ambient air conditions (Cook et al. 2015), but to our knowledge no one has examined the effects of polyphenol supplementation on exercise performance with trained athletes in high ozone conditions.

Therefore, the purpose of this study was to investigate the efficacy of 7 days of polyphenol supplementation on cycling time trial performance and respiratory function in healthy male adults exercising in ozone. We hypothesise there will be no difference in exercise performance or respiratory function between the polyphenol supplemented or control trials.

## Methods

### Participants

Thirteen healthy male cyclists (mean ± SD: age, 43.8 ± 12.38 years; height, 177.8 ± 7.1 cm; weight, 76.03 ± 7.88 kg; ${\dot{\text{V}}}$O_2max_ 4.12 ± 0.72 L min^−1^) volunteered to participate in this study. All subjects gave written informed consent after the experimental procedures, associated risks and potential benefits were explained and before the commencement of study participation. The study was approved by the Northern Health and Disability Ethics Committee (21/NTB/68), registered with the Australia New Zealand Clinical Trial Registry (ACTRN12622001198718) and conformed to the 2013 Declaration of Helsinki. An a priori analysis was conducted for sample size estimation using G*Power version 3.1.9.4 (Faul et al. 2009). The estimation was based on a meta-analysed effect of *d* = 0.45 for BC on exercise performance (Braakhuis, et al. 2020). Based on a repeated-measures ANOVA and between and within groups, *α* = 0.05 and power (1− β) = 0.8, yielded a required sample size of 12. Two subjects withdrew from the study after the first ozone exposure due to the respiratory symptoms experienced in the ozone environment, and one participant withdrew due to work commitments. Three subjects became infected with Covid during the trial. All three chose to return following their infection, but were required to complete their isolation period, and complete a minimum of four weeks of normal training before being readmitted to the study. Once readmitted, subjects had to complete the familiarisation trial again. The final sample size was, therefore, 10. Participants attended all testing sessions at the same time of day (± 90 min) and were required to refrain from strenuous physical activity and replicate any light training in the 24 h preceding each trial. Participants recorded their nutrition intake in the 24 h preceding the first trial and replicated this intake for all subsequent trials. Participants refrained from eating and only drank plain water for the two hours before each testing session commenced.

### Experimental design

**Fig. 1:**
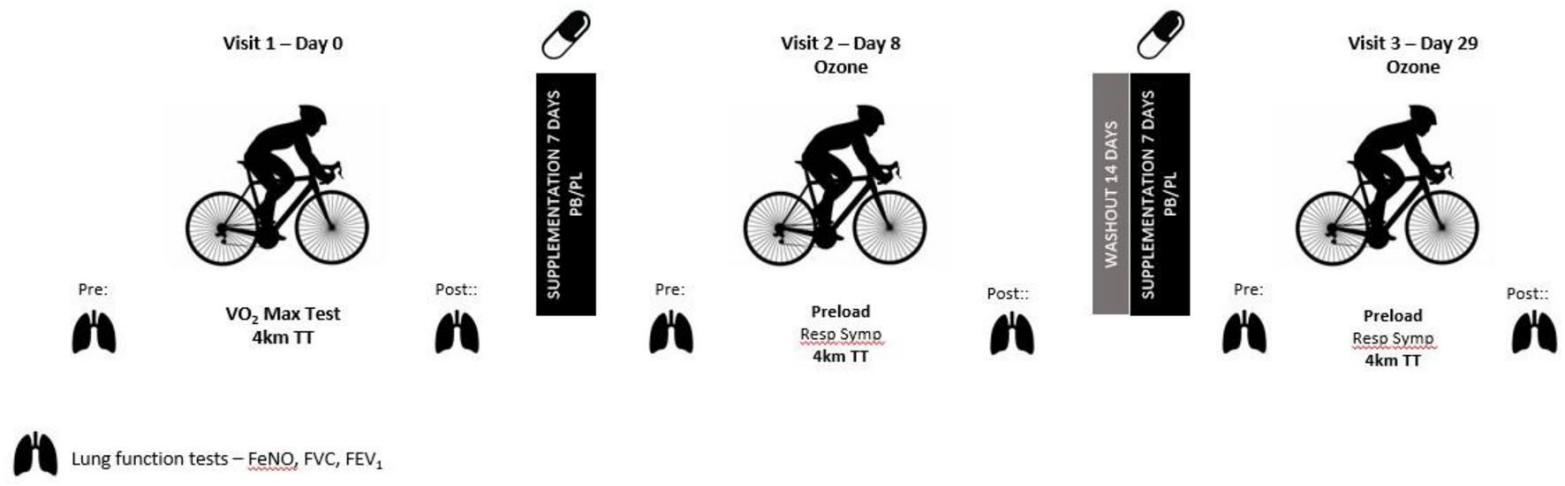
Study design and data collection points

The study was a randomised, double-blind, placebo-controlled crossover design. Participants visited the laboratory on three occasions. All subjects completed (i) an incremental test to exhaustion (${\dot{\text{V}}}$O_2max_) and familiarisation and (ii) two exercise testing trials in an ozone polluted environment following 7 days of supplementation of either placebo (PL) or blackcurrant (PB). During the initial visit to the laboratory, cyclists completed an incremental ramp test to determine their maximal oxygen consumption (${\dot{\text{V}}}$O_2max_) on a calibrated Velotron Dynafit Pro cycle ergometer (RacerMate Inc, WA, USA) using the company’s associated software package, followed by a 10-min rest and then a 4 km time trial (TT). The same cycle ergometer was used in all testing sessions. All components of the experimental procedure were included to familiarise participants with the test procedure and equipment. The incremental test and familiarisation were conducted in an environmentally controlled laboratory (temperature 19 ± 1º C: relative humidity 36 ± 6%) and ambient air conditions. The ozone trials were conducted in a sealed environmental chamber. Ozone was generated by silent discharge method (N50-C Ozone Generator) with a relay sensor (airQual, SM-70, Novozone, Auckland, NZ) to automatically maintain an ozone level of 0.25 ppm within ± 10%. There was a 14-d washout period between each supplement regime. All tests were supervised by the same researcher who was blinded to the experimental condition. A summary of the study design is shown in Fig. 1.

### Incremental test

On the first laboratory visit participants performed an incremental ramp test to determine maximal oxygen uptake (${\dot{\text{V}}}$O_2max_) and calculation of work-rate outputs (watts) for the preload/steady state protocol for the intervention trials. The cycle ergometer was adjusted to a position that resembled the set-up of the participant’s own racing bicycle and participants performed a 10-min warm-up at a self-selected sub-maximal intensity. Power output was increased to 100 W and increased continuously at a rate of 25 W.min^−1^ until the cyclist reached volitional exhaustion. Participants were required to remain seated throughout the test and maintain a cadence of 80 – 100 rpm. Indicators of achieving ${\dot{\text{V}}}$O_2max_ were volitional fatigue, a plateau in ${\dot{\text{V}}}$O_2_, a drop in cadence below 80 rpm, and/or a RER ≥ 1.1. During the incremental test, respiratory gases and heart rate were measured continuously with a calibrated metabolic system (Metamax, Cortex, Leipzig, Germany) using breath by breath mode. Peak power output (PPO) was defined as the average power sustained during the final 30-s of the test; ${\dot{\text{V}}}$O_2max_ was defined as the highest ${\dot{\text{V}}}$O_2_ measured over 30-s. Workloads for the preload protocol were calculated for each rider as 50%, 60% and 70% of the PPO achieved in the incremental test.

**Fig. 2:**
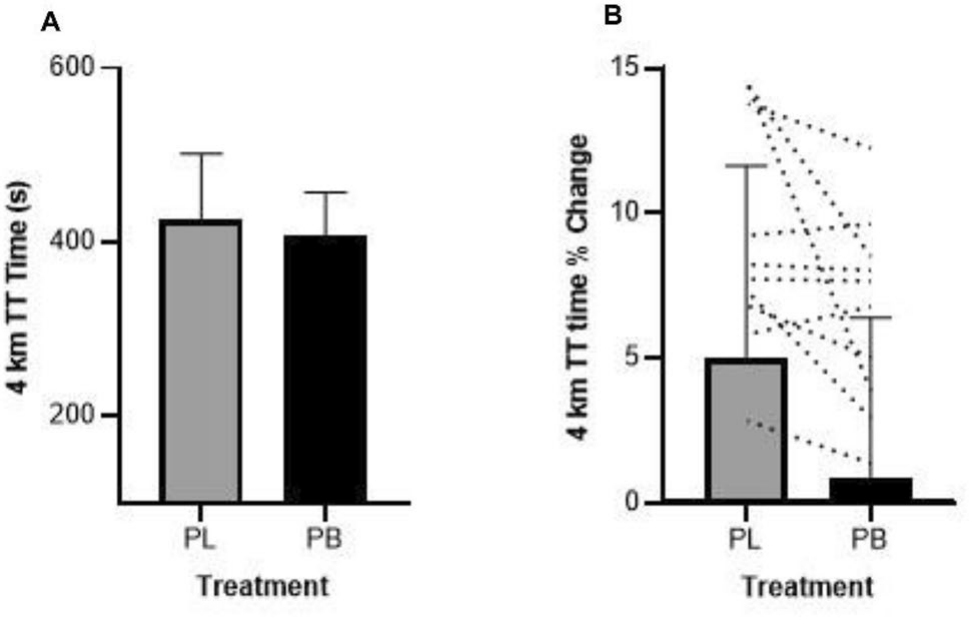
**A**—Mean ± SD 4 km TT time (s) in ozone following PB or PL supplementation for 7 days. **B** – Change in performance (%) relative to baseline following supplementation with PB or PL

### Experimental trial

All experimental arms were conducted in ozone (0.25 ppm). Subjects were randomly assigned in a crossover design to receive either PB or PL. Subjects consumed a commercially available product (Ᾱrepa™) containing freeze dried blackcurrant (PB) or PL for a period of 7 days prior to exercise testing. Subjects completed two separate intervention arms: PL + ozone cycling, and PB + ozone cycling. Each trial was separated by a 14-day washout period, after which subjects consumed their alternative treatment for 7 days before their final trial. Both the PB and PL were encapsulated into berry flavoured, purple gelatine capsules (The Capsule Guy, Adelaide, Australia) and dispensed into opaque unmarked bottles. The bottles were given to the researcher on the day of testing by an independent person for distribution to participants. The researcher remained blinded to the interventions throughout data collection. Supplement adherence was monitored by oversupplying participants with capsules and counting the returned capsules at each performance test.

The polyphenol blend powder (PB) contained a combination of freeze-dried NZ blackcurrant (94%), L-theanine (3%) and Pinus Radiata extract (3%) which provided 200 mg anthocyanins per 5g serve. PB was provided as a dose to provide 4.3 mg anthocyanins/kg body weight. The placebo was a manufactured berry flavoured powder matched for all nutritional composition, colour, and taste (Sensient, Auckland, New Zealand), but contained no phenolics, active ingredients or Vitamin C. To maintain the bioactivity of the phenolics, the PB capsules were made in batches close to testing. Supplements were allocated by an individual independent of the study, and neither the researcher nor subjects were aware of the intervention allocation.

Following 7 days of supplementation participants completed the experimental protocol. Participants were weighed when they arrived at the laboratory and fitted with measurement equipment. Participants then entered the environmental chamber and respiratory testing was performed and respiratory symptoms recorded. All cycling trials were performed on the ergometer described previously using the saddle and handlebar height recorded at the first visit. During all tests pulmonary gas exchange and ventilation were measured continuously using the metabolic system previously described. Participants performed a warm-up for 5 min, before completing a preload protocol consisting of 10 min at 50% PPO, 10 min at 60% PPO and 5 min at 70% PPO. Cycling load was automatically controlled by the ergometer. Ratings of perceived exertion (Borg RPE 6–20 scale) were obtained in the last 15 s of each stage during the preload. A 10 min rest followed the preload protocol and participants reported their respiratory symptoms. Participants were required to then complete a 4 km TT in the shortest time possible. Participants could only view their gearing and distance covered but were blinded to any other information to reduce potential pacing effects. Participants reported their RPE immediately after completion of the 4 km TT and then dismounted the ergometer. In a seated position within the chamber, participants completed a FeNO measure, and respiratory symptoms were assessed and another FeNo measurement completed (10-min post exercise). Maximal spirometry testing was then undertaken, once at 10 min post 4 km TT completion and another at 20 min post. Muscle oxygen saturation was measured from the onset of the preload until 15 min after the completion of the 4 km TT. All cycling data (time, distance, power, cadence, rpm, speed) was captured by the ergometer’s associated software package (RacerMate Inc, WA, USA). Participants were cooled with standing floor fans during the experimental trials and permitted to drink water ad libitum in the rest period and post the 4 km TT.

### Respiratory measurements

Fraction expired Nitric Oxide (FeNO) measurements were obtained using a FeNO analyser (NObreath, Bedfont Scientific Ltd, England. Concentration range 5 – 300 ppb, accuracy ± 5 ppb/ ± 10% of measured value, repeatability ± 5 ppb/ ± 10% of measured value) using the guidelines of the American Thoracic Society (ATS) (Dweik et al. 2011). In brief, subjects inhaled to total lung capacity and exhaled at a constant expiratory flow rate of 50 mL/s for approximately 6 s. All FeNO measures were obtained prior to spirometry testing as results have been shown to be affected by preceding maximal spirometry testing (Hoyte et al. 2018). Participants performed spirometry testing for forced vital capacity (FVC), forced expiratory volume (FEV_1_), forced expiratory flow (FEF _25–75%_), peak expiratory flow (PEF) according to the guidelines of the American Thoracic Society (Graham et al. 2019), using a digital spirometer interfaced to a computer (Medikro Pro, Kuopio, Finland. Volume range 0–14 L, flow range ± 14L/sec, flow accuracy ± 2% or 0.020 L/s, volume accuracy ± 2% or 0.05 L/s). Spirometry measures were repeated until two tests were within 150 mL limits of each other (Dweik et al. 2011; Graham et al. 2019), and the mean of the two highest qualified data values for FVC and FEV_1_ selected. Respiratory symptoms were assessed pre- and immediately post the preload protocol and post the 4 km TT using a visual analogue scale. Participants were asked to rate severity of cough (SOC), pain on deep inspiration (PDI), shortness of breath (SOB) and throat irritation (TI) on a five-point scale ranging from 0 (none) to 4 (most severe).

### Muscle oxygenation

Skeletal muscle oxygenation (%SmO_2_) of the vastus lateralis of the right leg was continuously measured using a near infrared spectroscopy (NIRS) sensor (Moxy monitor, Fortiori Design LLC, Minnesota, USA). The sensor was firmly attached at half the length of the vastus lateralis (greater trochanter to lateral knee joint space) in a black light blocking case to reduce light interference. The transmitted light was recorded at 0.5 Hz and received into proprietary software (PerfPro Software v 5.82.06, Hartware Technologies, Rockman, MI). Data were interpolated to 1-s intervals and averaged over every 60-s for the preload, and 15-s for the 4 km TT.

### Data analysis procedures and statistical analysis

Breath-by-breath data for ${\dot{\text{V}}}$O_2_, ${\dot{\text{V}}}$CO_2_, RER, ${\dot{\text{V}}}$E, BF and V_T_ from each test were examined for errant breaths and outliers (> 3 SDs from the mean) removed. The breath-by-breath data were averaged into 1-min intervals for each stage of the preload, and 2-min steady state data from each stage used for analysis of steady state cycling. The 4 km TT data (time, average power) was averaged by distance into 1 km distances. Breath-by-breath ${\dot{\text{V}}}$O_2_, ${\dot{\text{V}}}$CO_2_, RER data and mean power output (W) were averaged over the time for each km of the 4 km TT. Cycling efficiency could not be calculated at 70% PPO as RER values were > 1.0, and values therefore include unknown energetic contributions from anaerobic metabolism. Gross cycling economy of steady state cycling in the preload was calculated instead as WR/${\dot{\text{V}}}$O_2_ (W), where ${\dot{\text{V}}}$O_2_ of each stage was used as the measure of O_2_ cost and mean power output over the distance as the power output (WR). Skeletal muscle oxygenation was averaged into 1-min intervals from start (pre-preload) to 5 min post 4 km TT completion. The mean of 2-min steady state SmO_2_ was used as the value for each stage of the preload. Due to the inter-individual variability in FeNO measures, changes were expressed as percentage change from resting pre-exposure values.

Statistical analyses were conducted using GraphPad Prism version 9.3.0 for Windows (GraphPad Software, San Diego, California USA). The normality of data residuals was confirmed using the Shapiro–Wilk test prior to all analyses. Differences between treatments were determined using a one-way repeated measures analysis of variance (ANOVA), or via a two-tailed, two-way repeated measures ANOVA (treatment x time). Where significance was detected, Šídák’s multiple comparisons test post-hoc analysis was used to compare differences between treatments. Paired samples *t*-tests were used to compare change from pre or baseline between treatments. Fischer’s exact test of 2 × 2 contingency table (symptoms vs. no symptoms) was used to determine the association between treatment and each respiratory symptom (SOC, PDI, SOB and TI). Statistical significance was defined as *p* ≤ 0.05. The magnitudes of the standardised effects for the test measures were also determined using the Cohen effect size (*d*). Thresholds of 0.2, 0.5, and 0.8 for small, moderate, and large effects, respectively, were used in accordance with the recommendations of Cohen (1988). ES values < 0.2 were deemed trivial differences. Simple group statistics are shown as means ± SD, unless otherwise stated. Percentage (%) differences between treatments are reported as mean ± 95% confidence intervals (CI).

## Results

One participant was excluded from analysis as they were unable to complete the full preload protocol under the PL condition, therefore, nine subjects were included in the final analysis of all performance data.

### Steady state cycling

A summary of measured variables during steady state cycling in the preload is shown in Table 1. Heart rate, ${\dot{\text{V}}}$O_2_ (L min−1), ${\dot{\text{V}}}$CO_2_ (L min−1), ${\dot{\text{V}}}$E (L/min), breathing frequency (BF) tidal volume (V_T_), SmO_2_ responses, and RPE were not significantly different between PB and PL across the three intensities of the preload protocol (Table 2). At 60% PPO V_T_ was 2.75 ± 0.45 in PL and 2.65 ± 0.47 in PB (*p* = 0.06)). RER was lower at 70% PPO in the PB condition (*p* = 0.03). Cycling economy (W.L min−1) at fixed workloads were not different between the two conditions (*p* = 0.31).

**Table 1:** Metabolic responses, skeletal muscle oxygenation and cycling economy during the steady state preload cycling protocol (10-min at 50 PPO, 10-min at 60% PPO and 5-min at 70% PPO)

	50% PPO		60% PPO		70% PPO	
	Placebo	Polyphenol Blend	Placebo	Polyphenol Blend	Placebo	Polyphenol Blend
${\dot{\text{V}}}$O_2_ (L min−1)	2.79 ± 0.35	2.80 ± 0.47	3.27 ± 0.43	3.31 ± 0.56	3.72 ± 0.49	3.73 ± 0.66
${\dot{\text{V}}}$CO_2_ (L min−1)	2.59 ± 0.32	2.59 ± 0.47	3.11 ± 0.40	3.13 ± 0.53	3.76 ± 0.47	3.73 ± 0.70
Heart rate (bpm)	136 ± 11	135 ± 10	152 ± 12	154 ± 12	167 ± 12	167 ± 13
RER	0.93 ± 0.02	0.92 ± 0.03	0.95 ± 0.02	0.94 ± 0.02	1.01 ± 0.03	0.99 ± 0.02*
${\dot{\text{V}}}$E (L. min^−1^)	72.49 ± 7.81	70.60 ± 9.64	91.11 ± 12.26	92.31 ± 12.10	120.30 ± 14.51	116.93 ± 18.27
BF (min)	29.50 ± 6.12	28.95 ± 5.38	33.99 ± 6.91	35.54 ± 5.96	43.02 ± 8.36	41.82 ± 7.35
V_T_ (L)	2.53 ± 0.43	2.52 ± 0.52	2.75 ± 0.45	2.65 ± 0.47	2.86 ± 0.49	2.85 ± 0.52
SmO_2_ (%)	30.22 ± 5.28	31.07 ± 10.47	21.91 ± 5.66	21.87 ± 11.28	17.07 ± 5.52	17.46 ± 11.42
RPE	12 ± 1	12 ± 1	15 ± 1	15 ± 2	18 ± 1	18 ± 1
Cycling Economy (W.L min−1)	68.07 ± 5.63	69.91 ± 10.12	69.84 ± 5.65	66.93 ± 4.98	71.14 ± 4.55	70.84 ± 4.75

Data reported as mean ± SD from 9 subjects. *PPO* Peak power output, *V*_*T*_ Tidal volume, ${\dot{\text{V}}}$*E* ventilation, *BF* breathing frequency, *RER* respiratory exchange ratio, *RPE* rating of perceived exertion. * *p* < 0.05

**Table 2:** Mean power, ventilatory and gas exchange dynamics during 4 km TT cycling performance under ozone following 7-day supplementation with PB and PL

	1 km		2 km		3 km		4 km	
	PL	PB	PL	PB	PL	PB	PL	PB
Power (watts)	216.00 ± 95.89	244.74 ± 71.43**	232.75 ± 89.76	248.39 ± 74.55	242.99 ± 89.62	256.06 ± 78.29	277.43 ± 78.85	286.59 ± 74.64
${\dot{\text{V}}}$O_2_ (L min−1) ^*^	2.61 ± 0.67	2.80 ± 0.53	3.32 ± 0.80	3.51 ± 0.81	3.52 ± 0.69	3.70 ± 0.79	3.82 ± 0.69	3.95 ± 0.76
${\dot{\text{V}}}$CO_2_ (L min−1)	2.04 ± 0.66	2.15 ± 0.66	3.16 ± 0.90	3.24 ± 1.18	3.46 ± 1.02	3.49 ± 1.27	3.91 ± 0.84	3.81 ± 1.350
Heart rate (bpm)	138.78 ± 11.26	141.54 ± 13.96	155.16 ± 13.82	156.99 ± 13.46	161.66 ± 15.82	164.17 ± 13.51	169.35 ± 15.88	169.91 ± 12.96
RER	0.81 ± 0.06	0.80 ± 0.03	0.95 ± 0.06	0.96 ± 0.06	0.98 ± 0.05	0.98 ± 0.06	1.04 ± 0.05	1.02 ± 0.05
${\dot{\text{V}}}$E (L. min^−1^)	72.12 ± 14.90	75.66 ± 10.83	106.16 ± 19.96	106.80 ± 21.10	119.97 ± 25.21	121.01 ± 23.54	143.47 ± 21.17	140.63 ± 26.25
BF (min)	37.13 ± 8.39	38.62 ± 6.68	41.87 ± 7.53	42.42 ± 7.87	45.93 ± 10.46	47.14 ± 8.42	52.78 ± 10.21	54.27 ± 12.39
V_T_ (L)	1.99 ± 0.47	1.99 ± 0.38	2.61 ± 0.54	2.57 ± 0.53	2.71 ± 0.68	2.62 ± 0.57	2.81 ± 0.62	2.69 ± 0.62

Data reported as mean ± SD from 9 subjects. Significant main effect for treatment in ${\dot{\text{V}}}$O_2_ with PB supplementation compared to PL, * *p* = 0.01. ** Higher mean power output over the first km in PB, *p* = 0.01, 95% CI – 50.95 to − 4.74, *d* = 0.34. *RER* respiratory exchange ratio, *V*_*T*_ Tidal volume, ${\dot{\text{V}}}$E ventilation, *BF* breathing frequency

### 4 km cycling TT performance

Time to complete the 4 km TT with PB was 406.43 ± 50.29 s, and 426.20 ± 75.06 s with PL but were not significantly different (*p* = 0.09, 95% CI -43.47 to 3.92, *d* = 0.31, Fig. 2).

**Fig. 3:**
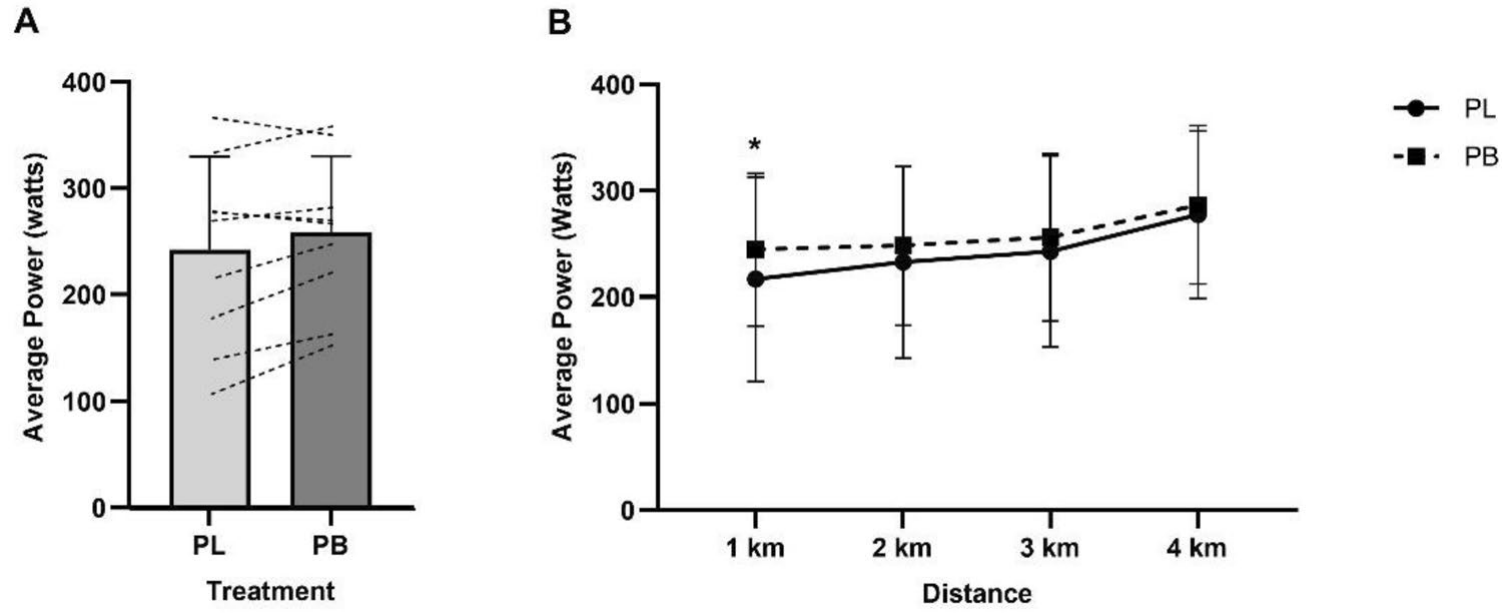
**A**—Mean ± SD power output (Watts) for the 4 km TT in ozone following PB or PL supplementation for 7 days. **B** – Average power output (Watts) per km of the 4 km TT. Power output in the first km was significantly higher following PB supplementation, * *p* = 0.01

Average power output in the 4 km TT was 258.95 ± 71.35 W following PB supplementation, compared to 242.52 ± 87.26 W with PL (16.43 ± 23.78 W, 95% CI 1.84 to 34.71, *p* = 0.07, *d* = 0.20). The average power output across the baseline 4 km TT was 274.56 ± 82.85 W. Analysis of mean power output between PB and PL in each km of the 4 km TT under ozone showed a significant main effect for distance (*p* < 0.01), and a significantly higher power output was observed in the first km of the TT following PB supplementation (− 27.85 W, 95% CI − 50.95 to − 4.75, *p* = 0.01, *d* = 0.33. Fig. 3). A summary of measured variables per km of the 4 km TT is shown in Table 2.

**Fig. 4:**
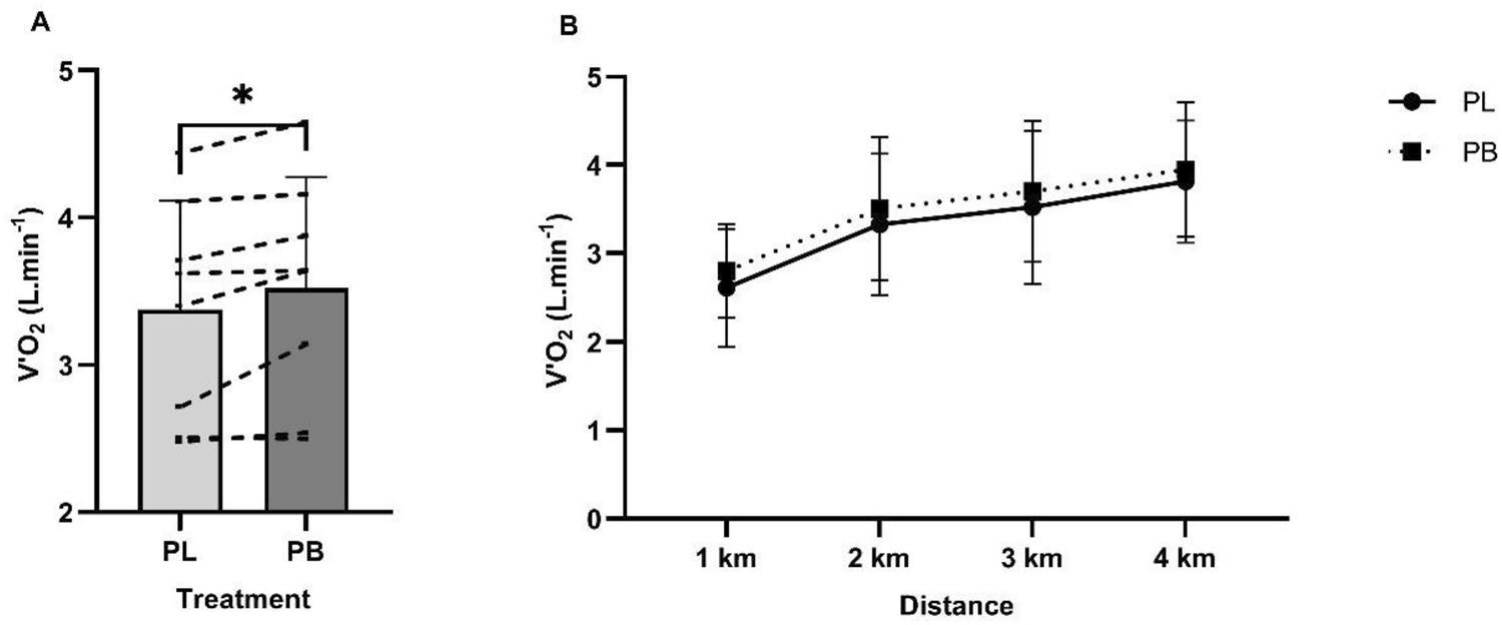
**A** Mean pulmonary oxygen uptake (${\dot{\text{V}}}$O_2_ L min−1) during 4 km TT cycling performance in ozone following 7 days supplementation of PB or PL. A significant difference in ${\dot{\text{V}}}$O_2_ during the 4 km TT observed with PB supplementation compared to PL, * *p* = 0.01. **B** – ${\dot{\text{V}}}$O_2_ per km of the 4 km TT

A significant treatment effect for ${\dot{\text{V}}}$O_2_ was found in the PB condition (− 0.17, 95% CI − 0.29 to − 0.05, *p* = 0.01, *d* = 0.24), with a higher mean ${\dot{\text{V}}}$O_2_ in the 4 km TT compared to PL (Fig. 4). No differences were observed in RPE, HR, ${\dot{\text{V}}}$CO_2_ or RER between the two conditions during the TT.

### Respiratory function

Ozone exposure resulted in significant decreases in FEV_1_, FVC, FVC/FEV_1_, FEF_25-75_ and PEF compared ambient air. FVC was reduced by 12.00 ± 13.81% in PL (*p* = 0.001) and 11.04 ± 9.21% in PB (*p* = 0.02) after cycling in ozone compared to a 6.94 ± 13.24% increase after exercise in ambient air conditions. FVC continued to decrease 20 min post the 4 km TT in the PL condition but improved slightly in the PB condition (PB − 9.03 ± 10.86%, PL − 12.73 ± 13.13%, *p* = 0.28, *d* = 0.28). Similarly, FEV_1_ decreased by 18.15 ± 13.49% with PL (*p* < 0.01) and 15.45 ± 10.48% with PB (*p* < 0.01) following exercise in ozone. FEV_1_ increased 5.36 ± 6.63% following exercise in ambient air. FEV_1_ measures improved in both PL (− 15.80 ± 16.50%) and PB (− 15.43 ± 12.33%, *p* = 0.99, *d* = 0.02) trials by 20 min post exercise. Lung function following ozone exposure was similar in PB and PL conditions. A significant time effect was found for FEF_25-75_ following ozone exposure. FEF_25-75_ decreased –22.03% ± 28.40% 15 min post cycling in ozone following PL supplementation and –36.56 ± 34.63% with PB supplementation (− 29.30, 95% CI − 4.72 to 33.79, *p* = 0.14, *d* = 0.46). At 20 min post exercise FEF25-75 decreased -34.77 ± 21.39% with PL treatment and − 29.19 ± 40.71% with PB (− 31.98, 95% CI − 24.83 to 13.68, *p* = 0.71, *d* = 0.17). The means of pre and post exercise spirometry measurements are summarised in Table 3. There was a decrease in FeNO (ppb) levels following exercise in ozone (*p* = 0.07, *d* = 0.49). Fisher’s exact test was used to determine if there was a significant association between supplementation and respiratory symptoms (SOC, PDI, SOB and TI). There was a statistically significant relationship between PB supplementation and severity of cough (*p* = 0.03, two tailed), with lower symptom scores compared to PL. No differences were observed for the other respiratory symptoms (Table 4).

**Table 3:** Respiratory responses and symptoms after cycling exercise in ozone (0.25 ppm) following PL or PB supplementation

	PRE		Post 1 (15 min)		Post 2 (20 min)	
	Placebo	Polyphenol Blend	Placebo	Polyphenol Blend	Placebo	Polyphenol Blend
FVC (L)	4.87 ± 0.98	4.82 ± 0.93	4.25 ± 0.90	4.28 ± 0.87	4.19 ± 0.82	4.37 ± 0.94
FEV_1_	3.75 ± 0.77	3.64 ± 0.62	3.01 ± 0.52	3.06 ± 0.53	3.10 ± 0.66	3.06 ± 0.63
FVC/FEV_1_	76.28 ± 6.45	76.42 ± 6.55	71.76 ± 8.43	72.35 ± 7.49	73.04 ± 9.30	71.48 ± 6.39
FEF_25-75_	3.20 ± 0.89	3.06 ± 0.65	2.43 ± 1.22	1.92 ± 1.15	1.91 ± 0.74	1.95 ± 1.16
PEF	8.04 ± 1.94	7.54 ± 1.51	6.70 ± 1.92	6.38 ± 1.50	6.49 ± 2.01	6.16 ± 1.64
FeNO	27.22 ± 20.90	27.33 ± 19.07	25.11 ± 21.24	23.00 ± 16.83	26.67 ± 21.03	26.00 ± 19.61

Data reported as mean ± SD. Pre testing was performed upon entry to the chamber containing ozone (< 2 min exposure time). FeNO—Fraction expired nitric oxide, FVC—forced vital capacity, FEV_1_—forced expiratory volume in 1 s, FEF_25-75_—forced expiratory flow over the middle one half of the FVC

**Table 4:** Respiratory symptom scores after cycling exercise in ozone (0.25 ppm) following PL or PB supplementation

	PRE		Post (15 min)		Post (20 min)	
	Placebo	Polyphenol Blend	Placebo	Polyphenol Blend	Placebo	Polyphenol Blend
SOC	2	0	10 *	7	12 *	6
PDI	2	1	19	20	19	14
SOB	0	5	22	20	21	16
TI	3	10	17	16	21	18

Respiratory symptom scores are the Σ of scores for 10 subjects. Pre testing was performed upon entry to the chamber containing ozone (< 2 min exposure time, *SOC*—severity of cough, *PDI*—pain on deep inspiration, *SOB*—shortness of breath, *TI*—throat irritation. * *p* = 0.03, two tailed

## Discussion

This study investigated the effects of 7 days of polyphenol blend supplementation at a standardised dose of 4.3 mg/kg anthocyanins on cycling performance in healthy males exercising under ozone conditions. We found no significant differences in measured variables across submaximal exercise intensities of 50%, 60% and 70% PPO in the preload. Furthermore, the 4 km TT time with PB supplementation (406 s) compared to PL (426 s) was not statistically different, but there was a small effect (*d* = 0.31). A significantly higher ${\dot{\text{V}}}$O_2_ was observed in the 4 km TT following PB supplementation, along with higher mean power in the first km compared to PL.

We did not statistically compare the 4 km TT times under ozone to the TT time in ambient air during familiarisation due to the differences in preload preceding the 4 km TT, but it is worth noting that the time in the PB treatment was only 1.86 ± 4.87% slower than ambient air performance compared to a decrease of − 6.18 ± 5.85% with PL. There were large individual changes in 4 km TT performance between the PB and PL conditions under ozone. Three subjects improved substantially, two subjects performed marginally worse, and four subjects had ~ equivalent 4 km TT times. Previous research has reported considerable individual variation in inflammation and lung function parameters as well as subjective symptomatic responses following ozone exposure (Hazucha et al. 2003, Mudway et al. 2001, Holz et al. 1999, Passannante et al. 1998). In the current study, supplementation with PB for 7 days resulted in a significant reduction in the severity of cough. We also observed lower scores in throat irritation, pain on deep inspiration and shortness of breath following PB treatment after both the preload protocol and the 4 km TT. The better performance in the subsequent 4 km TT in some subjects with PB could be related to a perceived lower stress due to reduced adverse respiratory symptoms.

Despite cross-sectional studies showing respiratory and pulmonary benefits from anthocyanin intake (Garcia-Larsen et al. 2015, Tan et al. 2014, Morton and Braakhuis 2021), the results from the present study failed to show any benefit from PB supplementation on lung function measures following acute ozone exposure. Significant decreases in FEV1, FVC, FEV/FEV1, and FEF_25-75_ were observed in subjects after cycling in ozone 15 min post exercise, and continued to decline at 20 min post. The greatest respiratory decreases were observed in FEF_25-75_ which decreased by as much as 30% in both PL and PB. FEF_25-75_ measures describe the flow from medium-to-small airways, and reductions in FEF_25-75_ reflects resistance and possible functional impairment of peripheral and small airways caused by inflammatory processes (Szefler et al. 2020). FEF_25-75_ is, however, non-specific, not uniformly reproducible and is dependent on FVC (Deepak et al. 2017). Measuring FeNO provides a non-invasive surrogate measure of airway inflammation and insight into inflammation in the distal airways. Nitric oxide (NO) in the lung promotes inflammation at higher concentrations as a result of the overactivity of oxidative pathways (Hoyte et al. 2018). The increase in FeNO levels with PL and PB in the present study at 10 min post exercise could indicate the onset of airway inflammation.

The ozone dose is an important factor in exposure studies with a cumulative dose determined by ozone concentration [ppb] x duration of exposure (min) x V’E (L/min) (Adams et al. 1981, Arjomandi et al. 2018). The inclusion of an incremental steady state protocol prior to a maximal effort 4 km TT was used to induce a fatiguing protocol relative to the rider’s individual ability and extend the ozone exposure to approximately 60 min. In addition to this, it allowed us to observe any perturbations to physiology and performance at fixed exercise intensities. Furthermore, the incremental increase in work rate would increase the inspiration of ozone. All measured variables were similar at work rates of 50% and 70% PPO. Breathing frequency increased, tidal volume decreased, and gross cycling economy were lower with PB treatment at 60% PPO. Collectively these shifts indicate a higher respiratory cost to produce the workload, and a higher BF and lower V_T_ a shallower breathing pattern, which typically indicates lower perfusion rates. A reduction in V_T_ has been observed in previous work with ozone exposure (Adams 1987).

The current study is novel in its attempt to use polyphenols supplementation to improve athletic performance in a polluted environment. The lack of other studies examining antioxidant supplementation, ozone and exercise performance makes comparisons difficult. In addition, the lack of clear underlying physiological mechanisms means that any reasons for improved performance are speculative and based on assumptions from studies in ambient air. To our knowledge one other controlled study has utilised an antioxidant intervention to determine the effects on exercise performance in ozone. Gomes et al. (2011) performed a randomised, double-blind crossover to determine if supplementation of Vitamin C (500 mg/day) and vitamin E (100 IU/day) for two weeks would improve 8 km running performance in a hot (31°C), humid (70%), ozone polluted (0.10 ppm) environment. Despite changes to plasma and nasal lavage antioxidant status, no effect on running performance was found, despite positive correlations between antioxidant concentration and improvements in running time.

A major limitation of the current study is the reduced sample size which was unfortunately caused by subject drop out beyond our control. The loss of participants compromised our final statistical analysis. A larger sample size would have aided clearer effects of PB supplementation, or lack thereof, more clearly on exercise performance under ozone. Another limitation is we did not control for subjects normal dietary polyphenol intake, choosing instead to supplement anthocyanins with the usual diet. This may have resulted in higher polyphenol and anthocyanin consumption in some subjects. However, previous work has estimated the mean consumption of anthocyanins from dietary sources to be only 43 – 67 mg/day (Copetti et al. 2022). Finally, while we prescribed the polyphenol powder based on the anthocyanin content, we acknowledge the commercial powder also contained other extracts (L-theanine and pine bark) which may have affected the findings.

## Conclusion

Supplementation with a polyphenol blend at a dose of 4.3 mg/kg.bw anthocyanins for 7 days resulted in a small but non-significant increase in 4 km cycling performance. Oxygen consumption during the TT was also significantly greater with polyphenol treatment. Finally, the severity of cough experienced when exercising in ozone was significantly reduced with supplementation. Further studies with larger sample size are required to confirm the results of this study and to determine the underlying mechanisms responsible for any potential benefits.

## Data Availability

The data that support the findings of this study are available from the corresponding author, L. C. Morton, upon reasonable request.
